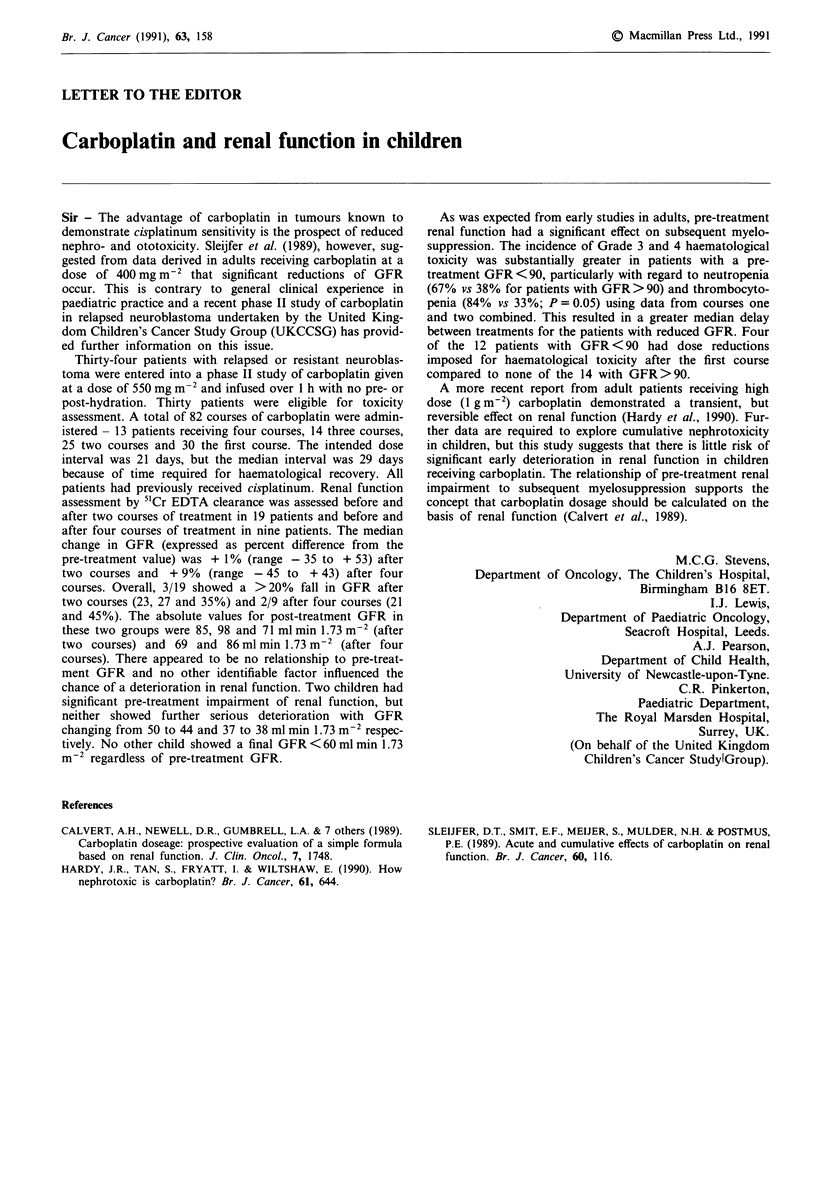# Carboplatin and renal function in children.

**DOI:** 10.1038/bjc.1991.34

**Published:** 1991-01

**Authors:** M. C. Stevens, I. J. Lewis, A. J. Pearson, C. R. Pinkerton


 © Macmillan Press Ltd., 1991

### LETTER TO THE EDITOR

### Carboplatin and renal function in children

**Sir** – The advantage of carboplatin in tumours known to demonstrate *cis*platinum sensitivity is the prospect of reduced nephro- and ototoxicity. Sleijfer *et al.* (1989), however, suggested from data derived in adults receiving carboplatin at a dose of 400 mg $m^{-2}$ that significant reductions of GFR occur. This is contrary to general clinical experience in paediatric practice and a recent phase II study of carboplatin in relapsed neuroblastoma undertaken by the United Kingdom Children's Cancer Study Group (UKCCSG) has provided further information on this issue.

Thirty-four patients with relapsed or resistant neuroblastoma were entered into a phase II study of carboplatin given at a dose of 550 mg $m^{-2}$ and infused over 1 h with no pre- or post-hydration. Thirty patients were eligible for toxicity assessment. A total of 82 courses of carboplatin were administered – 13 patients receiving four courses, 14 three courses, 25 two courses and 30 the first course. The intended dose interval was 21 days, but the median interval was 29 days because of time required for haematological recovery. All patients had previously received *cis*platinum. Renal function assessment by $^{51}$Cr EDTA clearance was assessed before and after two courses of treatment in 19 patients and before and after four courses of treatment in nine patients. The median change in GFR (expressed as percent difference from the pre-treatment value) was + 1% (range – 35 to + 53) after two courses and + 9% (range – 45 to + 43) after four courses. Overall, 3/19 showed a >20% fall in GFR after two courses (23, 27 and 35%) and 2/9 after four courses (21 and 45%). The absolute values for post-treatment GFR in these two groups were 85, 98 and 71 ml min 1.73 $m^{-2}$ (after two courses) and 69 and 86 ml min 1.73 $m^{-2}$ (after four courses). There appeared to be no relationship to pre-treatment GFR and no other identifiable factor influenced the chance of a deterioration in renal function. Two children had significant pre-treatment impairment of renal function, but neither showed further serious deterioration with GFR changing from 50 to 44 and 37 to 38 ml min 1.73 $m^{-2}$ respectively. No other child showed a final GFR < 60 ml min 1.73 $m^{-2}$ regardless of pre-treatment GFR.

As was expected from early studies in adults, pre-treatment renal function had a significant effect on subsequent myelosuppression. The incidence of Grade 3 and 4 haematological toxicity was substantially greater in patients with a pre-treatment GFR < 90, particularly with regard to neutropenia (67% *vs* 38% for patients with GFR > 90) and thrombocytopenia (84% *vs* 33%; $P = 0.05$) using data from courses one and two combined. This resulted in a greater median delay between treatments for the patients with reduced GFR. Four of the 12 patients with GFR < 90 had dose reductions imposed for haematological toxicity after the first course compared to none of the 14 with GFR > 90.

A more recent report from adult patients receiving high dose (1 g $m^{-2}$) carboplatin demonstrated a transient, but reversible effect on renal function (Hardy *et al.*, 1990). Further data are required to explore cumulative nephrotoxicity in children, but this study suggests that there is little risk of significant early deterioration in renal function in children receiving carboplatin. The relationship of pre-treatment renal impairment to subsequent myelosuppression supports the concept that carboplatin dosage should be calculated on the basis of renal function (Calvert *et al.*, 1989).

M.C.G. Stevens,
Department of Oncology, The Children's Hospital,
Birmingham B16 8ET.
I.J. Lewis,
Department of Paediatric Oncology,
Seacroft Hospital, Leeds.
A.J. Pearson,
Department of Child Health,
University of Newcastle-upon-Tyne.
C.R. Pinkerton,
Paediatric Department,
The Royal Marsden Hospital,
Surrey, UK.
(On behalf of the United Kingdom
Children's Cancer Study Group).

**References**

CALVERT, A.H., NEWELL, D.R., GUMBRELL, L.A. & 7 others (1989). Carboplatin doseage: prospective evaluation of a simple formula based on renal function. *J. Clin. Oncol.*, **7**, 1748.

HARDY, J.R., TAN, S., FRYATT, I. & WILTSHAW, E. (1990). How nephrotoxic is carboplatin? *Br. J. Cancer*, **61**, 644.

SLEIJFER, D.T., SMIT, E.F., MEIJER, S., MULDER, N.H. & POSTMUS, P.E. (1989). Acute and cumulative effects of carboplatin on renal function. *Br. J. Cancer*, **60**, 116.